# Immune Response to Vaccination in Patients with Psoriasis Treated with Systemic Therapies

**DOI:** 10.3390/vaccines8040769

**Published:** 2020-12-16

**Authors:** Andrea Chiricozzi, Paolo Gisondi, Francesco Bellinato, Giampiero Girolomoni

**Affiliations:** 1 Dermatologia, Dipartimento Scienze Mediche e Chirurgiche, Fondazione Policlinico Universitario A. Gemelli IRCCS, 00168 Rome, Italy; 2 Dermatologia, Università Cattolica del Sacro Cuore, 00168 Rome, Italy; 3 Section of Dermatology and Venereology, Department of Medicine, University of Verona, 37129 Verona, Italy; paolo.gisondi@univr.it (P.G.); francesco.bellinato@gmail.com (F.B.); giampiero.girolomoni@univr.it (G.G.)

**Keywords:** psoriasis, vaccination, vaccine, biologics, methotrexate, cyclosporine, apremilast, dimethyl fumarate

## Abstract

Psoriasis is a chronic inflammatory skin disease usually treated with immunomodulatory/immunosuppressive agents. The use of these agents has been associated with an increased susceptibility to infections. Vaccination might represent a critical aspect in the management of patients with psoriasis treated with immunomodulatory/immunosuppressive therapies. This narrative review aimed to provide an overview on the immune response to vaccines in subjects treated with systemic agents used to treat patients with moderate to severe psoriasis. Publications appearing in PubMed, Scopus, and ISI–Web of Knowledge database were selected using Medical Subject Headings key terms. Overall, published data confirmed that vaccination with attenuated live vaccines during therapy with immunomodulatory/immunosuppressive therapies should be avoided. For nonlive vaccines, a more favorable safety profile of biologic agents compared to conventional systemic agents is described as the humoral response to vaccines is in general well-preserved. Treatment with cyclosporine and methotrexate is associated with lower antibody titers to vaccines, and thus these agents are better discontinued during vaccination. In contrast, treatment with biological agents is not associated with lower antibody response and can thus be continued safely.

## 1. Introduction

Psoriasis is a chronic inflammatory skin disease affecting 2–4% of the population in Western countries [1]. Advances in the understanding of the complex pathogenic mechanism led to the identification of different therapeutic targets that are selectively neutralized by so-called biologic agents. Drugs used in the treatment of moderate to severe psoriasis include TNF-α blockers (adalimumab, certolizumab, infliximab, and etanercept) as well as interleukin (IL)-23p19 (risankizumab, tildrakizumab, and guselkumab), IL-12/IL-23p40 (ustekinumab), and IL-17 blockers (secukinumab, ixekizumab, and brodalumab). Small molecule systemic agents include conventional (i.e., methotrexate, dimethyl fumarate, and cyclosporine) as well as a novel (namely apremilast) molecule. Some of these agents are also indicated in children—in particular, TNF-α inhibitors and ustekinumab are labeled for use in children older than 4 years and 12 years, respectively.

Because the use of systemic immunomodulatory/immunosuppressive medications is associated with both an increased susceptibility to infections and a higher risk of complications to some vaccine-preventable infections, vaccination may have a relevant role in preventing or reducing this risk [2,3,4]. In addition, vaccination is a common practice in both children and older adults. Thus, it is very important to know whether systemic antipsoriatic agents alter the immune response to vaccines. This is even more important now that extremely large populations will be soon vaccinated for the Sars-2 Covid19 infection.

## 2. Materials and Methods

A search on three databases, MEDLINE (PubMed), SCOPUS, and ISI–Web of Knowledge, (ResearchGate) was performed using the following Medical Subject Headings (MeSH) key terms: “psoriasis” AND “vaccination”, “vaccines”, “immunosuppressive agents”, or “biologics”. Further papers were also selected and considered from the reference list of the above retrieved articles. The search included articles in English language only, published in the past 10 years (2010–2020), and screened by title and abstract, and subsequently evaluated by reading the full text.

## 3. Type of Vaccines

Conventionally, two main groups of vaccines can be identified based on their composition being constituted by either attenuated live pathogens or nonlive pathogens (Table 1). Live vaccines, constituted by attenuated natural pathogens, induce a durable and rapid humoral response [5]. As they mimic the natural infection, these vaccinations are usually associated with symptoms and risk of viral transmission, persistence, and active infection. Thus, they are contraindicated in immunocompromised patients. Nonlive vaccines (also known as inactivated or inert vaccines) are considered safer as they contain the killed pathogen, purified pathogen antigens, or inactivated toxins. Although they usually need to be repeated overtime (boosters), they are advantageous as they do not contain the active pathogen, and their storage and delivery are easier. In addition to these two classes of vaccines, an innovative type, defined as genetic-based vaccines, is being designed against SARS-CoV-2 infection. These new vaccines act as inactivated, nonlive vaccines, as they do not contain nonreplicating pathogens. Based on the conventional vaccine classification, they are included in the nonlive vaccine type but conversely to the protein-based class, they are developed as a nonreplicating viral vector, mRNA or DNA, that is encoded and transcripted into viral proteins by the host cells, generating an immune response once expressed [6]. Vaccines using the viral-vector technique transfer genetic information of the viral pathogen in a less harmful, engineered, nonreplicating virus, whereas vaccines using the DNA and mRNA platform consist of naked nucleic acids or, more recently, are encapsulated into a nanoparticle carrier [6,7]. This method shows some advantages, including rapidity in vaccine production and versatility as the same platform can be used to design different vaccines for different pathogens. Nevertheless, the viral vector platform, conversely to mRNA- or DNA-based vaccines, owns the disadvantage of a pre-existing immunity against the viral vector that can potentially limit vaccination effectiveness [6,7]. Though clinically unproven, the mRNA-based platform was selected for developing anti-SARS-CoV-2 vaccines and they are thought to behave like nonlive, inactivated vaccines, leading experts to consider them very promising because of their potential efficacy and safety profile.

## 4. Systemic Antipsoriatic Therapies and Vaccination

Prior to starting an immunomodulatory/immunosuppressive therapy, vaccination status should be checked. Commonly, live vaccines are considered contraindicated if administered during treatment with systemic immunomodultant/immunosuppressive therapies, while concomitant administration of nonlive vaccines, according to national vaccination schedule, may be considered [8,9]. In high-risk situations wherein the potential risk of infection is considered to outweigh the risk related to the administration of live vaccines, vaccination could be considered according to an infectious disease consultant. Live vaccine can be administered either 2–4 weeks before starting therapy or, after temporary therapy interruption of 1 to 3 months, in order to boost an adequate immune response. On the contrary, nonlive vaccines may be given concomitantly to immunomodultant/immunosuppressive therapies. The immunogenicity of nonlive vaccines appeared to be preserved during the use of biologic agents, whereas methotrexate or other conventional systemic therapies may significantly impair humoral response to vaccines. In fact, protective immune response to vaccination is maintained concomitantly with the inhibition of IL-17, IL-23/IL-12, or TNF, despite some evidence suggesting a detrimental effect of their blockade. IL-17-deficient mice models showed an impaired antibody production, suggesting that IL-17 may promote B cell activation [10,11]. However, this effect on antibody secretion is thought to be indirect, as in vitro experiments demonstrated that IL-17, IL-17F or IL-17E (IL-25) alone was not able to induce antibody production by B cells [12]. In addition, B cells cocultured with IL-17-deficient CD4+ T cells showed a preserved antibody production, suggesting that Th17 cell-derived IL-17 is not required. Because IL-23 is the main regulator of IL-17 expression, its role on plasma cell activity might be considered dispensable. On the contrary, IL-12 stimulates IFN-γ expression in Th1 cells, which in turn enhances the immunoglobulin class-switching into IgG class [13]. Despite the inhibition of IL-12 by ustekinumab, no evidence of antibody production impairment was observed. TNF inhibition was associated with both plasma cell and memory B cell reduction after influenza vaccination [14,15]. However, no evidence of meaningful reduction of vaccine-induced antibody production in human beings treated with TNF inhibitors in monotherapy was reported.

### 4.1. Conventional Systemic Medications

#### 4.1.1. Cyclosporine

Cyclosporine is shown to reduce antibody titers postvaccination. Studies on cyclosporine-treated transplanted patients showed lower humoral response to vaccine stimulation for influenza, keyhole limpet hemocyanin, tetanus, and hepatitis B Virus [16,17].

#### 4.1.2. Dimethyl Fumarate

A single open-label, multicenter, nonrandomized study provided evidence that dimethyl fumarate treatment did not reduce T-cell dependent or independent humoral immune responses [18]. This study included 71 patients with relapsing-remittent multiple sclerosis, treated with either 240 mg dimethyl fumarate, administered twice daily for at least 6 months (*n* = 38) or nonpegylated interferon for at least 3 months (*n* = 33), assessing humoral response to tetanus-diphtheria, meningococcus C, and pneumococcal vaccination. Tetanus-diphtheria toxoid (recall antigen), conjugated meningococcal C polysaccharide (neoantigen), and pneumococcal vaccine mounted a comparable immune response (response was defined as a ≥2-fold increase from pre- to postvaccination titer) between the two treatment groups [18]. A positive immune response, defined as a ≥4-fold increase in antibody titer to the three vaccines, was achieved in both treatment groups, though no negative control represented by healthy or untreated subjects was included [18].

#### 4.1.3. Methotrexate

A consistent body of evidence showed lower response to vaccination during methotrexate therapy. The negative impact of methotrexate on humoral response was detected when used both in monotherapy and in combination with biologic agents. Reduced antibody titers were observed after exposure to various nonlive vaccines, including seasonal influenza virus and pneumococcus [19,20,21,22,23,24,25,26]. Studies on rheumatoid arthritis patients demonstrated a significant and substantial enhancement of the postvaccination humoral response to trivalent seasonal influenza vaccine if methotrexate was discontinued for 2–4 weeks [27,28]. Nevertheless, because a 4-week discontinuation was associated with an increased risk of disease worsening, a shorter period of discontinuation (2 weeks) improved the immunogenicity of seasonal influenza vaccination minimizing the risk of disease flaring [27,28].

### 4.2. Novel Synthetic Medications

#### Apremilast

No information about the impact of apremilast therapy on humoral response induced by nonlive vaccines is currently available.

### 4.3. Biologic Agents

There is consensus among experts, and national and international guidelines in avoiding vaccination with attenuated live vaccines during therapy with biologic agents, independently from their target and mechanism of action. Contrarily, nonlive vaccines can be administered in patients undergoing therapy with biologics as their selective immune-modulation does not affect vaccine-induced antibody production (Table 2).

#### 4.3.1. TNF-α Inhibitors

Vaccine immunogenicity has been tested only marginally in patients with psoriasis. Most studies have been performed in rheumatoid arthritis (RA) patients by measuring immune response to influenza and pneumococcal vaccines, but these patients represent a very good model, and thus data on these patients are very likely to apply also to patients with psoriasis. In general, vaccination during anti-TNF-α monotherapy is considered effective. Some data suggested decreased responses to antigen stimulation when TNF inhibitors are combined with methotrexate [26,29]. Albeit nonlive vaccines can be safely administered during anti-TNF-α therapy, especially in monotherapy, as the immunization to vaccines is not affected, suboptimal vaccine use during anti-TNF-α therapy has been reported [30,31].

##### Adalimumab

Adalimumab was positively tested in patients with RA, demonstrating a preserved and effective immunization to both pneumococcal and influenza vaccines [32]. In a substudy enrolling 64 patients, vaccine response meant as a 2-fold increase in pneumococcal antibody titer to any of four pneumococcal antigens was similar in patients treated with adalimumab or placebo. Overall, 22.5% to 52.5% of adalimumab-treated patients and 7.7% to 46.2% of placebo-treated patients responded positively to pneumococcal vaccine challenge at 4 weeks postvaccination [32].

A randomized, double-blind, placebo-controlled, multicenter Phase IV trial, assessing antibody production in response to the standard 23-valent pneumococcal vaccine and the influenza trivalent virus vaccination, assigned 115 RA patients to placebo and 111 to adalimumab treatment [32]. Following pneumococcal vaccination, percentages of patients achieving a vaccine response were similar between the two groups (37.4% and 40.4%, respectively). Across both groups, the percentage of patients who achieved a ≥2-fold titer increase in ≥3 of 5 pneumococcal antigens was higher in the group without protective antibody titers at baseline [32]. Univariate analyses demonstrated that concomitant methotrexate use (*p* < 0.0001), concomitant disease-modifying antirheumatic drugs (DMARDs) use (*p* < 0.044), and protective antibody titers at baseline (*p* < 0.0001) significantly reduced the response rate to pneumococcal vaccine in the adalimumab group compared to the placebo group [32]. The number of patients achieving a ≥2-fold increase in ≥3 of the 5 pneumococcal titers was lower in patients treated with adalimumab and methotrexate (10/55, (18.2%)) compared to patients receiving placebo and methotrexate (17/59, (28.8%)) or adalimumab alone (27/44, (61.4%)). Similarly, a lower number of patients achieved the same response if treated with adalimumab and other DMARDs (hydroxychloroquine and/or leflunomide) compared to patients receiving placebo and other DMARDs (2/17, (11.8%) vs. 11/32, (34.4%)) or adalimumab alone (35/82, (42.7%)).

Percentages of patients achieving protective antipneuomococcal antibody titers were similar in both treatment groups (adalimumab: 85.9%, placebo: 81.7%), while protective anti-influenza antibody titers were detected in 94.5% and 98% of patients treated with placebo and adalimumab, respectively [32]. Considering high anti-influenza antibody titers (≥4-fold increase), a smaller, although not statistically significant percentage of patients receiving adalimumab achieved this vaccine response compared to placebo (51.5% vs. 63.3%), likely due to the presence of pre-existing protective anti-influenza antibodies [32]. There was contrasting data on the response rate to influenza vaccination obtained with concomitant use of methotrexate: a reduced response was observed, but neither methotrexate nor other DMARDs significantly lowered humoral response in adalimumab-treated patients compared to placebo associated with methotrexate (29/55, (52.7%) vs. 33/59, (55.9%)) or other DMARDs (11/17, (64.7%) vs. 18/32, (56.3%)).

Another RA-based study assessed the effects of adalimumab therapy on the immunogenicity of pneumococcal and influenza vaccination in patients with RA [33]. It revealed that adalimumab did not affect response to pneumococcal vaccination. Though one-third of patients responded to none or only 1 of the 7 serotypes tested, the poor result was likely due to the disease itself [33].

##### Certolizumab Pegol

In a single-blind placebo-controlled Phase 3 trial, RA patients (*n* = 110) treated with certolizumab pegol showed similar antibody response to pneumococcal polysaccharide vaccine and influenza vaccine, compared to placebo (*n* = 114) [34]. Patients receiving certolizumab pegol concomitantly with methotrexate had a lower humoral response compared with patients receiving certolizumab pegol alone, though the clinical meaning of this reduced antibody production is not known [34]. Both placebo patients and certolizumab pegol-treated patients receiving concomitant methotrexate had lower pneumococcal vaccine responses compared to patients in monotherapy (certolizumab pegol vs. placebo with concomitant methotrexate: 44.4% vs. 50.0%; certolizumab pegol vs. placebo in monotherapy: 80.0% vs. 89.3%). Similarly, the response rates to influenza vaccination were reduced with the concomitant use of methotrexate in patients treated with either placebo or certolizumab pegol (certolizumab pegol vs. placebo with concomitant MTX: 45.8% vs. 50.9%; certolizumab pegol vs. placebo in monotherapy: 70.4% vs. 84.6%) [34].

##### Etanercept

Etanercept was not reported to interfere with the humoral response to pneumococcal polysaccharide antigen challenge. The pneumococcal vaccine response was maintained in patients affected by psoriatic arthritis undergoing therapy with etanercept (*n* = 94), compared to placebo-treated patients (*n* = 90). This patient population derived from a randomized, double-blind, placebo-controlled trial designed to examine the efficacy of etanercept (25 mg twice weekly) [35]. In the trial, 67% of patients were able to mount an adequate immune response to pneumococcal vaccination challenge with a 2-fold increase in titer to 2 or more antigens, while a 4-fold increase to 2 or more antigens was detected in 47% of patients [35]. A 2-fold increase of antibody titer was similar between the group receiving etanercept (in which the proportion of responders ranged from 35% to 62% across the 5 antigens) and the placebo group (in which the proportion of responders ranged from 40% to 62%). The combined use of methotrexate and advanced age was a predictor of a poor response to vaccination. In particular, patients receiving methotrexate were less likely to respond to antigen challenge than those not receiving methotrexate, with a lower proportion of patients classified as vaccine responders (27%–42% vs. 47%–79%) [36].

##### Infliximab

A substudy deriving from a Phase 3 trial included 90 adult patients affected by rheumatoid arthritis and treated with either infliximab and methotrexate, or placebo and methotrexate [37]. The combination of infliximab and methotrexate did not interfere with the immune response to vaccine, obtaining an effective 2-fold increase in titers to polyvalent pneumococcal vaccine, similar to the control group treated with methotrexate plus placebo [37]. Measures of vaccine response in psoriasis patients undergoing therapy with infliximab have not been published.

#### 4.3.2. p40IL-12/IL-23 Inhibitor: Ustekinumab

No dedicated studies investigating the impact of ustekinumab on the immunogenic response to vaccine have been performed. Nevertheless, a substudy of the Phoenix 2 trial evaluated the immunogenicity of pneumococcal and tetanus vaccines in 60 psoriasis patients treated with ustekinumab in comparison with 56 psoriasis patients who were not treated with systemic therapies [38]. More than 90% of both treatment groups (96.6% of ustekinumab-treated patients vs. 92.6% of controls) responded with a ≥2-fold increase in antibody levels to ≥7 of 14 serotypes, and similar results were obtained for the tetanus vaccine as well, obtaining a ≥4-fold-increase in 84.7% of ustekinumab- treated patients and in 77.8% of controls [38].

#### 4.3.3. IL-17 and IL-17 Receptor Antagonists

##### Brodalumab

No data related to the impact of brodalumab on humoral response to nonlive vaccines are currently available in psoriasis patients.

##### Ixekizumab

A randomized, open-label, parallel-group Phase 1 study assessed immune response to tetanus and pneumococcal vaccines in healthy subjects exposed to ixekizumab [39]. Vaccination was performed alone (*n* = 42 healthy subjects) or in combination with 160 mg ixekizumab subcutaneously injected 2 weeks prior to vaccination and 80 mg ixekizumab on the day of vaccination (*n* = 41). Response to vaccines was evaluated 4 weeks postvaccination [39]. Adequate response to tetanus was defined by the presence of antitetanus antibodies ≥1.0 IU and a ≥1.5-fold increase if baseline was ≤1.0 IU, or a ≥2.5-fold increase if baseline was >1.0 IU [39]. Immunization with ixekizumab resulted noninferior to the control group based on a prespecified noninferiority margin of 40% for the difference in proportions of responders at 4 weeks postvaccination between ixekizumab and the control groups. For the tetanus vaccine, adequate response was seen in 52.6% and 51.2% of subjects in the ixekizumab and control groups, respectively [39]. Adequate response to pneumococcal vaccination was defined as a ≥2-fold increase from baseline in antipneumococcal antibodies against >50% of the 23 serotypes. Response was seen in 89.5% and 90.2% of subjects in the ixekizumab and control groups, respectively [39]. No data related to the impact of ixekizumab on humoral response to nonlive vaccines are currently available in psoriasis patients.

##### Secukinumab

Different clinical trials tested humoral response to influenza and meningococcal vaccines during secukinumab monotherapy [38,39,40]. No impairment of the immunogenic response to vaccines has been described in adult subjects treated with secukinumab. In particular, an open-label, parallel-group, randomized single-center study evaluated immunogenicity to inactivated trivalent subunit influenza virus and conjugate group C meningococcal vaccine in 50 healthy subjects receiving a single 150 mg dose of secukinumab or no treatment [40]. Antibody responses to vaccinations were comparable in both groups, with ≥4-fold increased titers to influenza virus vaccination of 20/25 (80%) for both groups and similarly, for meningococcal vaccination, obtaining ≥4-fold increased titers in 76% (19/25) and 72% (18/25) of secukinumab-treated subjects and control group, respectively [40]. Another study including 30 subjects—17 patients affected by psoriatic arthritis or ankylosis spondylitis and 13 healthy controls—confirmed that secukinumab did not suppress the humoral immune response to the influenza virus vaccine [41]. The immunogenic response to the trivalent vaccine consisting of an inactivated A/Michigan/45/2015 (H1N1) pdm09-like virus, an A/Hong Kong/4801/2014 (H3N2)-like virus, and a B/Brisbane/60/2008-like virus was preserved in patients treated with secukinumab compared to untreated healthy subjects (median values of increase: 4.6-fold and 4.0-fold, respectively, for anti-H1N1; and 3.7-fold and 5.3-fold, respectively, for anti-B Ab). Both groups presented a poor response against H3N2, with a <1.5-fold increase [41]. Overall, no significant differences in the proportion of patients who responded to the vaccine were found between the two subgroups. Notably, the immunogenic response to the same trivalent influenza virus vaccine (H3N2, H1N1 and B/Victoria) was also preserved if secukinumab was combined with synthetic DMARDs [42]. Humoral response of healthy subjects was compared to 32 patients suffering from psoriatic arthritis and treated with secukinumab monotherapy or combined with conventional synthetic disease-modifying drugs, mostly methotrexate (in about one-third of cases, 10 patients) [42]. Similar rates of seroprotection after vaccinations were found in secukinumab-treated patients and in healthy controls. Secukinumab, even when combined with DMARDs, did not impair antibody production as titers were high and similar among patients and controls (for H1N1 81% vs. 93%, respectively; for H3N2 90% vs. 100%, respectively; for B 100% in both groups) [42].

#### 4.3.4. p19IL-23 Inhibitors

The p19IL-23 inhibitors guselkumab, risankizumab, and tildrakizumab constitute the most recently marketed class of biologic agents for the treatment of moderate to severe psoriasis. No data on the response to live or inactive vaccines are available for any of these agents.

### 4.4. Special Focus on Vaccines against SARS-COV-2

Rapid spread of the SARS-COV-2 pandemic caused vaccination strategies. Several potential candidate vaccines being rapidly developed are in clinical evaluation. Considering the crucial role of the SARS-CoV-2 spike (S) glycoprotein in virus attachment, entry, and induction of neutralizing antibodies, S protein is being widely used as a target for vaccine development. Based on advances in techniques for vaccine design, inactivated, live-vectored, nucleic acid, and recombinant COVID-19 vaccines are being developed and tested for their efficacy. Phase 3 clinical trials are underway or will soon begin for several of these vaccines. Assuming that clinical efficacy is shown for one or more vaccines, safety is a major aspect to be considered before deploying such vaccines to the public. The current review focuses on the recent advances in recombinant COVID-19 vaccine research and development, and associated issues.

## 5. Conclusions

The introduction of biologic agents in the treatment of psoriasis profoundly changed long-term management because of their much higher efficacy and safety over conventional systemic agents. Biologic agents were demonstrated to be advantageous also in the context of vaccination, a safety aspect that is currently becoming prominent. In this respect, the use of conventional systemic compounds, such as methotrexate and cyclosporine, is associated with an impairment of the humoral response to vaccines as they reduce antibody production, lowering protective antibody titers. Biologic agents, determining a selective immune-modulation, seem to not interfere with antibody production, preserving the humoral response to vaccines. Surprisingly, even if nonlive vaccines can be safely administered during treatment, vaccination programs are apparently not successful among patients affected by psoriasis. Indeed, vaccination coverage rates are reported to be low in patients with moderate to severe psoriasis. A noninterventional, cross-sectional study performed in Germany detected 28% of psoriasis patients treated with systemic therapies to be vaccinated, in line with the general German resident population above 18 years [43]. A similar vaccination rate was reported among psoriatic arthritis patients [44]. This vaccination coverage was relatively low considering that these patients are meant as a risk population because of their immunocompromised status and psoriasis-related comorbidities. Indeed, the vaccination rate in the psoriasis population was significantly lower than the rate reported in chronically ill patients, including chronic respiratory, cardiovascular, and malignant diseases as well as liver/kidney diseases and diabetes (season 2007/08: 44%, 95% CI 42–46; and season 2008/09: 42%, 95% CI 40–44). Compared to patients with RA, psoriatic patients were less likely to receive a flu vaccine, and this difference was magnified in people younger than 50 years [45]. This poor vaccine distribution among psoriatic patients was negatively affected by patient motivations and concerns about safety because of the occurrence of psoriasis onset or worsening concomitantly with vaccination. In particular, cases of psoriasis flares or new onset have been reported within three months following immunization with Bacillus Calmette-Guérin, seasonal influenza virus, or tetanus/diphtheria toxins [46,47,48,49].

Physician reluctance or neglect might represent an additional negative factor impacting vaccine distribution as about 40% of patients reported getting vaccinations at their own request, while influenza vaccination was suggested by 43.4% and 6.6% of general practitioners and dermatologists, respectively [43]. Because dermatologists own a profound knowledge of both the disease and therapy (especially for biologic agents), they should represent the most relevant vaccine-related information source for psoriasis patients and actively encourage patients with psoriasis to undergo vaccination.

## Figures and Tables

**Table 1:** Type of vaccines categorized by their composition and formulation.

Type of Vaccine	Example of Pathogen
Live attenuated	Poliomavirus (oral polio vaccine) Rotavirus Measles morbillivirus Mumps orthorubulavirus Rubella virus Varicella-zoster virus Tuberculosis (bacillus Calmette-Guerin) Yellow fever virus Cholera yes or not Typhoid fever yes or not
Inactivated (killed pathogen)	Pertussis (whole-cell) Inactivated polio virus
Subunit	Pneumococcus (PCV-7, PCV-10, PCV-13) Pertussis (acellular pertussis) Hepatitis B virus Haemophilus influenzae type B herpes zoster (recombinant herpes zoster vaccine)
Toxoid (inactivated toxins)	Tetanus toxoid Diphtheria toxoid
Genetic-based vaccines (mRNA, DNA, viral vector)	Influenza A/H10N8 (mRNA platform) Influenza A/H7N9 (mRNA platform) SARS-CoV-2 (mRNA platform)

**Table 2:** Details about biologics and vaccination.

Class of Agent	Therapeutic Agent	Molecular Structure	Therapeutic Target	Evidence on Immunogenicity Post-Vaccination
ANTI-TNF	Adalimumab	Human monoclonal antibody	TNFα	Yes
	Certolizumab pegol	Pegylated humanized antibody Fab’ fragment	TNFα	Yes
	Etanercept	Dimeric fusion protein consisting of the extracellular ligand-binding portion of the human p75 TNF receptor linked to the Fc portion of human IgG1	TNFα	Yes
	Infliximab	Chimeric monoclonal antibody	TNFα	Yes
ANTI-IL-12/IL-23	Ustekinumab	Human monoclonal antibody	p40IL-12/IL-23	Yes
ANTI-IL-17	Brodalumab	Human monoclonal antibody	IL-17RA	No
	Ixekizumab	Human monoclonal antibody	IL-17A	Yes
	Secukinumab	Human monoclonal antibody	IL-17A	Yes
Anti-IL-23	Guselkumab	Human monoclonal antibody	p19IL-23	No
	Risankizumab	Humanized monoclonal antibody	p19IL-23	No
	Tildrakizumab	Humanized monoclonal antibody	p19IL-23	No
